# *Salmonella* Typhimurium DT104, Italy

**DOI:** 10.3201/eid1208.050968

**Published:** 2006-08

**Authors:** Amy Cawthorne, Pasquale Galetta, Marco Massari, Anna Maria Dionisi, Emma Filetici, Ida Luzzi

**Affiliations:** * Istituto Superiore di Sanità, Rome, Italy

**Keywords:** Keywords: Salmonella Typhimurium, DT104A, Italy

To the Editor: The recent article by Helms et al. described the distribution of Salmonella enterica serovar Typhimurium definitive page type 104 (DT104) infections in 29 countries from 1992 to 2001 (*1*). Results from Italy were not presented because routine phage typing was not performed before 2001. Since 2002, circulation of S. Typhimurium phage types has been monitored by the laboratory-based surveillance system Enter-net Italia, which was coordinated by Istituto Superiore di Sanità as part of the European network for the surveillance of foodborne infections (*2*). From 2000 to 2004, S. Typhimurium accounted for ≈40% of all human Salmonella isolates each year. Since 2002, ≈20% of the S. Typhimurium isolates were identified as DT104, and all had a pentavalent resistance pattern (resistance to ampicillin, chloramphenicol, streptomycin, sulfonamides, and tetracycline) (*3*). Although the results reported by Helms et al. (*1*) refer to a different period (1992–2001), the Italian data are similar to those from many other countries in northern and western Europe.

According to the Colindale scheme for phage typing (4 and L.R. Ward, pers. comm.), numerous distinguishable DT104 subtypes can be identified as DT104 A, B, C, H, and L. Most (90%) S. Typhimurium DT104 strains isolated during the last 2 years belonged to subtype DT104L.

Emergence of phage subtype DT104A was identified in June 2004 during an outbreak of salmonellosis in Rome. This subtype had never been previously identified in Italy. All DT104A isolates were susceptible to the Enter-net panel of antimicrobial drugs (*2*), a feature unusual for S. Typhimurium (*5*). A total of 63 cases were confirmed; 61 were from Rome, and 2 were from a neighboring region. All isolates had similar pulsed-field gel electrophoretic profiles when analyzed with the Salm-gene protocol (*6*). Since the outbreak, 1 additional human isolate of DT104A was identified from a resident of the same neighboring region. This isolate was also susceptible to the panel of antimicrobial drugs. A fermented pork salami was epidemiologically implicated as the vehicle of infection. No microbiologic evidence was found because no food samples were available when the outbreak was recognized.

The incidence of DT104 in Italy has remained stable from 2002 through 2004. However, emergence of subtype DT104A during a recent outbreak highlights the need for subtyping in identifying communitywide outbreaks and in monitoring changing subtype patterns.
